# Ovarian Torsion in Polycystic Ovary Syndrome: A Potential Threat?

**DOI:** 10.3390/biomedicines11092503

**Published:** 2023-09-10

**Authors:** Iason Psilopatis, Christos Damaskos, Nikolaos Garmpis, Kleio Vrettou, Anna Garmpi, Efstathios A. Antoniou, Athanasios Chionis, Konstantinos Nikolettos, Konstantinos Kontzoglou, Dimitrios Dimitroulis

**Affiliations:** 1 Department of Obstetrics and Gynecology, University Erlangen, Universitaetsstrasse 21-23, 91054 Erlangen, Germany; iason.psilopatis@alumni.charite.de; 2 Second Department of Propedeutic Surgery, Laiko General Hospital, Medical School, National and Kapodistrian University of Athens, 11527 Athens, Greece; nikosg22@hotmail.com (N.G.);; 3 Nikolaos Christeas Laboratory of Experimental Surgery and Surgical Research, Medical School, National and Kapodistrian University of Athens, 11527 Athens, Greece; 4 Renal Transplantation Unit, Laiko General Hospital, 11527 Athens, Greece; 5 Department of Cytopathology, Sismanogleio General Hospital, 15126 Athens, Greece; kliovr1@gmail.com; 6 Department of Gynecology, Laiko General Hospital, Medical School, National and Kapodistrian University of Athens, 11527 Athens, Greece; annagar@windowslive.com (A.G.);; 7 Obstetric and Gynecologic Clinic, Medical School, Democritus University of Thrace, 68110 Alexandroupolis, Greece

**Keywords:** polycystic, ovary, syndrome, adnexal, ovarian, torsion, emergency

## Abstract

Polycystic ovary syndrome (PCOS) constitutes the most prevalent endocrine disorder in women of reproductive age worldwide. Given the increased risk of ovarian torsion in the presence of large ovarian cysts, polycystic ovarian syndrome could be regarded as one of the most significant risk factors for ovarian and/or adnexal torsion in cases of significantly enlarged ovaries. The aim of the present review is to investigate, for the first time, the association between polycystic ovarian syndrome and ovarian torsion. We performed a review of the literature using the MEDLINE and LIVIVO databases in order to find relevant studies. By using the search terms “polycystic ovarian syndrome” and “ovarian torsion”, we were able to identify 14 studies published between 1995 and 2019. The present work constitutes the most up-to-date, comprehensive literature review focusing on the risk of ovarian/adnexal torsion in patients with polycystic ovaries. Ovarian/adnexal torsion seems to be a feared complication in patients with polycystic ovary syndrome. Acute lower abdominal pain in patients with known polycystic ovaries represents the most common symptom, while diagnostic assessment almost always incorporates transvaginal ultrasound and computer tomography or magnetic resonance tomography scans. In case of suspected torsion, emergency laparoscopy with ovarian or adnexal detorsion seems to be the standard therapeutic approach with a view to restitute the interrupted blood supply. In cases of repeated ovarian/adnexal torsions, ovariopexy or ovariectomy/adnexectomy had to be discussed with the patient in the context of risk recurrence minimization.

## 1. Introduction

The most prevalent endocrine disorder in women of reproductive age worldwide is polycystic ovary syndrome (PCOS) [1]. Symptoms typically begin during adolescence and range from insulin resistance and related health issues to irregular menstrual cycles and skin conditions [2]. After ruling out other endocrinological conditions, PCOS is diagnosed in adults depending on the fulfilment of at least two of the Rotterdam criteria. The presence of two of the following is required by the Rotterdam criteria: hyperandrogenism, oligo-ovulation and/or anovulation, or ovarian volume 10 mL (enlarged) and/or presence of multiple cystic follicles in one or both ovaries (polycystic ovary) on ultrasound [3]. Laboratory tests are crucial for confirming hyperandrogenism and ruling out alternative endocrinological conditions like hyperprolactinemia or thyroid dysfunction in addition to a thorough medical history and scholastic clinical examination [4]. The evaluation of mental health and quality of life, as well as metabolic screening and monitoring, should occur both at the initial visit and at regular intervals because women with PCOS are at a high risk of developing serious comorbidities [5]. Modifications to the patient’s lifestyle should always be a part of the standard therapeutic protocol for PCOS patients. More specifically, the foundation of non-pharmacological approaches is made up of weight loss, caloric restriction, specialized diets, and physical activity [5,6,7]. The primary goals of the pharmaceutical approach for PCOS patients who do not want to become pregnant are the control of menstrual cycle irregularities and hyperandrogenism, the handling of comorbidities, and the enhancement of quality of life. Biguanides may be supplementarily applied in addition to combined oral contraceptives and lifestyle changes with a view to ameliorating menstrual cycle abnormalities, weight, and metabolic outcomes [8]. Combined oral contraceptives or progestins are the first-line medications of choice for menstrual irregularities and/or hyperandrogenism. Antiandrogens are used to treat hirsutism and alopecia in women who cannot tolerate combined oral contraceptives [9]. Letrozole or clomiphene should be administered to PCOS patients who intend to become pregnant in order to trigger ovulation [10]. Notably, it has been also demonstrated in the literature how supplementation of vitamin D, inositols, or alpha lipoic acid could improve reproductive outcomes in PCOS patients [11,12,13]. Due to the ovulatory dysfunction associated with PCOS, affected patients usually also develop luteinized unruptured follicle syndrome [14]. This particular category of patients usually needs to resort to in vitro fertilization treatment methods to get pregnant, alongside the psychosocial implication in their infertile pathway [15].

When the ovary twists over the ligaments that support it in the adnexa, the process known as ovarian torsion takes place [16]. Adnexal torsion is the term used when the fallopian tube frequently twists along with the ovary [16]. An ovarian mass with a diameter of 5 cm or more is the main risk factor for ovarian torsion [17]. The mass makes it more likely that the ovary will rotate around the axes of the two ligaments that keep it suspended [18]. This torsion prevents arterial inflow and, eventually, venous outflow [18]. Females of all ages can experience torsion but childbearing-age women are more likely to experience it [16]. The infundibulopelvic and utero-ovarian ligaments, which serve as the ovary’s supporting ligaments, are torn when the ovary twists over them. Swelling and blood flow obstruction result from this. Initial obstruction of the venous outflow is followed by interruption of the arterial inflow as a result of increased swelling, which results in ovarian necrosis, infarction, hemorrhage, and possibly peritonitis [19]. Because the sigmoid colon is located in the left pelvis, the right side has been seen more frequently than the left; this is thought to be a result of the increased space in the right pelvis [18].

A complete metabolic panel, a serum human Chorionic Gonadotropin (hCG), and a complete blood count should all be performed in terms of laboratory testing. If the torsion is causing hemorrhage, leukocytosis or anemia may be profound. Given that pregnancy increases the risk of torsion, hCG is particularly significant. The lab values will typically be normal in torsion despite these generalized laboratory abnormalities [16]. The preferred imaging study is ultrasound with Doppler. It is necessary to perform both transvaginal and transabdominal ultrasounds. The sensitivity of ultrasound for ovarian torsion depends on a variety of elements, including the expertise of the physician and the anatomy of the patient. Free fluid or the whirlpool sign, which is thought to be caused by vascular pedicle twisting in the cross-section, may also be present. It is important to measure blood flow in comparison with the ovary on the opposite side. Because the ovaries have two blood supplies, a complete lack of flow is not required for symptoms to exist [20]. Because ovarian torsion may not be present at the time of ultrasound, this test alone cannot exclude it. Although they are frequently used to rule out other abdominal pathologies like acute appendicitis, computer tomography (CT) and magnetic resonance tomography (MRI) are not typically used to diagnose ovarian torsion [21,22].

Direct observation of a rotated ovary during surgery allows for the final determination of the diagnosis of ovarian torsion. For this reason, the patient must always undergo surgical evaluation if clinical suspicion persists, despite relatively normal lab results and ultrasound imaging. The surgeon must determine whether the ovary is viable before attempting to salvage it in females who are of reproductive age [16]. Surgery should typically be performed laparoscopically and with direct visualization of the twisted ovary. Visualization is primarily used to assess viability. Blood flow may be compromised in an enlarged, dark ovary with hemorrhagic lesions; however, the organ is frequently still salvageable [18]. More than 90% of patients who underwent detorsion had functional ovaries after the procedure. This was determined by the way the adnexa appeared on ultrasound, including the development of follicles on the ovaries. So, the preferred course of treatment is surgery with adnexal sparing [23]. Rarely, the surgeon may decide to perform a salpingo-oophorectomy if the ovary appears necrotic and gelatinous beyond repair. If a benign cyst is present, the surgeon may also perform a cystectomy. Salpingo-oophorectomy is the recommended course of treatment if the cyst appears to be cancerous or if the patient is postmenopausal [16].

**Figure 1:**
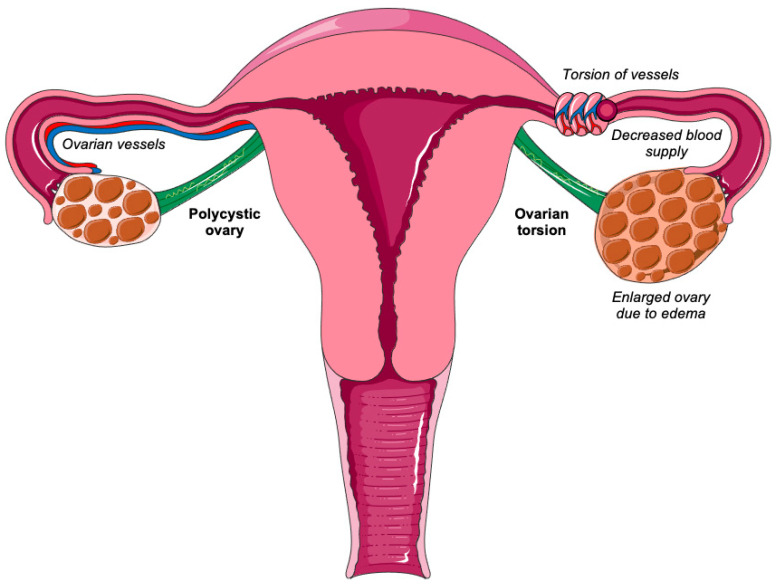
Ovarian torsion in polycystic ovary syndrome.

Given the increased risk of ovarian torsion in the presence of large ovarian cysts, polycystic ovarian syndrome could be regarded as one of the most significant risk factors in cases of significantly enlarged ovaries (Figure 1). However, so far, no review article has been published on the association of polycystic ovarian syndrome and ovarian torsion. Thus, the aim of the present work is to investigate, for the first time, the association between polycystic ovarian syndrome and ovarian torsion.

## 2. Literature Research

**Figure 2:**
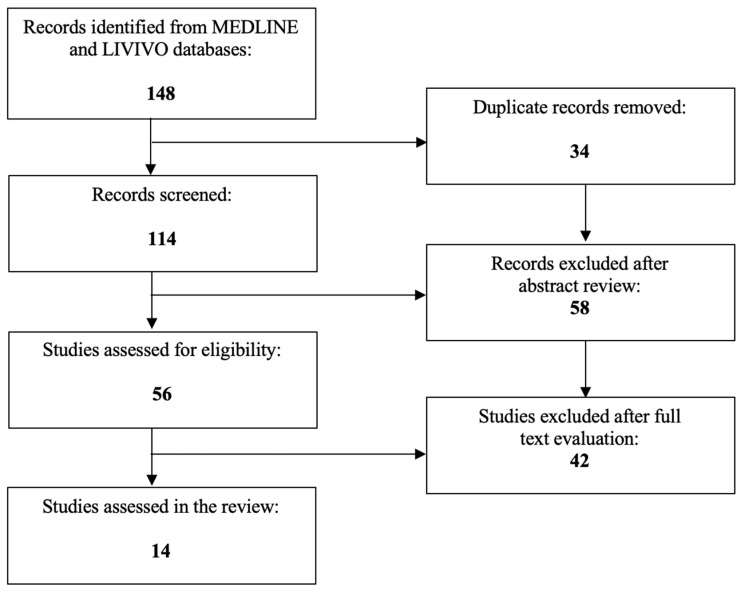
PRISMA flow diagram visually summarizing the screening process.

We performed the literature review by using the MEDLINE and LIVIVO databases. Original research articles written in the English language that clearly reported on the association between polycystic ovarian syndrome and ovarian torsion were included in the data analysis. Studies centering purely on the role of ovarian cysts in the absence of polycystic ovarian syndrome or that did not explicitly specify the presence of polycystic ovarian syndrome were excluded. By employing the search terms “polycystic ovarian syndrome” and “ovarian torsion”, we identified a total of 114 (duplicate records removed) articles published between 1966 and 2023. After the abstract review, 58 records were discarded in the initial selection process. The full texts of the remaining 56 publications were assessed. A total of 14 relevant case reports meeting the inclusion criteria and published between 1995 and 2019 were selected for the final literature review. The aforementioned selection process is schematically depicted in Figure 2.

## 3. Ovarian Torsion in Polycystic Ovary Syndrome

By scholastically searching the literature, we were to identify a total of twelve cases of adnexal torsion in patients with polycystic ovaries aged between 8 and 37 years old (mean age at diagnosis: 25 years old).

Asch et al. commented that torsion can be difficult to diagnose in patients with polycystic ovary syndrome who undergo ovarian stimulation, given the already enlarged ovaries at baseline [24].

Giulini et al. presented the case of a 31-year-old woman with polycystic ovary syndrome and right adnexal torsion during pregnancy after an oocyte in vitro maturation and intracytoplasmic sperm injection cycle. Two days after embryo transfer, the patient presented with right lower abdominal pain and leukocytosis. Transvaginal ultrasound revealed an enlarged right ovary, alongside normal bilateral ovarian vascular flow. Due to symptom deterioration, an explorative laparoscopy was preformed, identifying a twisted right adnexa with an ischemic ovary. After successful laparoscopic detorsion with preservation of adnexa, the patient had an uneventful postoperative course and delivered a healthy infant at 40 weeks of gestation [25].

Furthermore, Gonçalves et al. reported the case of a 31-year-old woman with polycystic ovary syndrome and recurrent right adnexal torsion. The patient first presented with acute right pelvic pain and the sonographic constellation of acute adnexal torsion. After emergency laparoscopy, the enlarged polycystic right ovary was successfully detorsed and the ovary and tube regained their normal colors. Three months later, the patient experienced a similar set of symptoms, with the radiologic results being once again consistent with recurrent torsion of the right adnexa. The diagnosis was confirmed by a second laparoscopic exploration, and the utero-ovarian ligament was sutured with a non-absorbable suture. The patient started having milder, more persistent right abdominal discomfort two months after the second surgery. This time, MRI revealed an enlarged, edematous right ovary with ipsilateral abnormal ovarian enhancement, suggesting a subacute process. The second recurrence of right adnexal torsion was confirmed by a subsequent laparoscopy. Two fixation points were used to perform a different oophoropexy between the right adnexa and the ipsilateral round ligament. The right adnexa torsion returned after the patient had been followed up for a full year without experiencing any pertinent symptoms. The decision to perform a unilateral right adnexectomy was met after thorough explanation and the patient’s informed consent. The procedure went smoothly, and the recovery time was typical. An ovary that was necrotic and hemorrhagic was found during anatomopathological analysis. Consequently, the patient exhibited no further signs of a recurrence of contralateral ovarian torsion [26].

Moreover, Hiei et al. described the case of a 22-year-old patient with polycystic ovaries and a six-hour history of right lower abdominal pain upon hospital admission. MRI revealed bilateral enlarged ovaries with a right twisted and thickened peduncle, a finding that ultimately led to the decision to perform an exploratory laparotomy with detorsion of the twisted ovary and drilling of the bilateral ovaries [27].

Additionally, Tay et al. announced the case of a 31-year-old woman with polycystic ovarian syndrome who experienced chronic, severe pelvic pain and presented with a sonographically suspected hydrosalpinx and a small amount of free fluid. An exploratory laparoscopy revealed that the woman had isolated left fallopian tube torsion. Following successful detorsion, the patient reported symptom absence at the six-week follow-up [28].

Matsuoka et al. presented a case of polycystic ovaries with bilateral adnexal torsion occurring asynchronously during a natural cycle of a 37-year-old woman suffering from lower left abdominal pain. Left ovarian edematous swelling, alongside ventral movement to the uterus, was detected on ultrasound and MRI, thus rendering an urgent laparoscopic adnexectomy necessary. Nine months after this primary operation, she began to experience right lower abdominal pain. The patient underwent a second emergency laparotomy, after suspected right adnexal torsion was discovered by ultrasonography. The right ovary had partial polycystic changes, was 7 cm in size, and was twisted 540 degrees counterclockwise. After the right adnexectomy, the patient had an uneventful postoperative course and received hormone replacement therapy [29].

Furthermore, Murakami et al. published their case report on a 21-year-old nulliparous woman with polycystic ovaries who complained of right lower abdominal pain and was diagnosed with ischemic edema of the right ovary by MRI. A necrotic right ovary that was purplish-black in color and had undergone a 540° torsion around the utero-ovarian ligament was discovered during an emergency laparoscopy, justifying the decision to conduct a successful right salpingo-oophorectomy [30].

Moreover, Obut et al. announced the impressive case of left ovarian torsion of a 21-year-old female patient with polycystic ovarian syndrome for the seventh time in a row. At the fifth and sixth laparoscopic surgeries, ovarian fixation was attempted in addition to detorsion but failed. The authors used a different technique: folding the utero-ovarian ligament, which had folded on itself due to the recurrence of ovarian torsion following ovarian fixation. The ovary and the proximal portion of the round ligament, which was next to the uterus, were both fixed to the distal portion of the utero-ovarian ligament. The postoperative period was uneventful, with the blood flow to both ovaries remaining normal at follow-up [31].

In addition, Pryor et al. reported on a 29-year-old patient with polycystic ovary syndrome who developed ovarian hyperstimulation syndrome during an IVF-ET cycle and experienced a torsion of the right tube and ovarian pedicle. Cyst drainage was performed, and a pelviscopic technique was used in a detorsion attempt; however, a laparotomy was ultimately necessary to complete the detorsion. The distal tube’s color returned to normal after the pedicle had been rotated 360 degrees. Although the tube was edematous, no palpable thrombus was present. Following surgery, the patient experienced symptoms of ileus. About 40 h after the initial procedure, an exploratory laparotomy was conducted. Non-purulent ascitic fluid and a necrotic right ovary without recurrent torsion were discovered. During the right salpingo-oophorectomy, a thrombus was seen in the vessels as the pedicle was being cut [32].

Shah et al. suggested screening premenarchal girls with ovarian torsion, who do not show obvious ovarian pathology, for ultrasound and biochemical signs of polycystic ovary syndrome. In cases where polycystic ovary syndrome is present, oral contraceptives may be used to reduce ovarian volume, taking into account the patient’s age and pubertal development [33].

Sheizaf et al. reported the case of an 8-year-old girl with polycystic ovaries that underwent a laparoscopic untwisting after initially presenting with right adnexal torsion. Four additional laparoscopies were required over the course of the next three years to treat left adnexal torsions. Torsion returned despite twice undergoing bilateral utero-ovarian ligament plication. The left ovary was ultimately fixed to the sidewall just below the pelvic brim [34].

Shi et al. investigated a 34-year-old woman with polycystic ovarian syndrome and a history of infertility who presented with abdominal pain and persistently enlarged ovaries. Ultrasonography and serum tests were used to assess the enlarged ovaries. The patient was treated by diagnostic laparoscopic surgery with detorsion and drainage followed by Gonadotropin-Releasing Hormone agonist (GnRH-a) therapy. Five days after the procedure, the patient was released from the hospital without any notable complications. After receiving GnRH-a injections every month for three consecutive months, both ovaries were nearly back to normal [35].

Shukunami et al. published the case of a 19-year-old nulligravida with polycystic ovaries who was diagnosed with twisted right adnexa and an unexplained anemia with subsequent shock. Emergent laparotomy revealed torsion of a right paraovarian tumor together with a right polycystic ovary. The finding of necrotic adnexa justified the conduction of a right salpingo-oophorectomy. After an uneventful recovery without blood transfusion, the patient was discharged in a stable condition [36].

Simsek et al. announced the case of a 20-year-old woman with polycystic ovaries who presented with right adnexal torsion for the sixth time. In the third and fifth laparoscopies, she experienced two unsuccessful ovarian fixation attempts. At elective surgery one month after the last detorsion operation, a combined ovarian fixation method was employed in order to fix the ovary to the pelvic side wall and shorten the utero-ovarian ligament. At follow-up, there was no evidence of recurrent adnexal torsion [37].

Table 1 summarizes the characteristics of the aforementioned cases.

## 4. Discussion

Complete or partial rotation of the adnexal supporting organ with ischemia is referred to as ovarian torsion, to which females of all ages are susceptible. In between 2% and 15% of patients who undergo surgical treatment for adnexal masses, ovarian torsion develops [16]. An ovarian mass is the main risk in the context of ovarian torsion. According to data from the World Health Organization (WHO), polycystic ovary syndrome affects about 116 million women worldwide [38]. As such, enlarged polycystic ovaries could be regarded as one of the major threatening factors leading to ovarian torsion and potentially adnexal necrosis. Nonetheless, no review article to date has been published on the correlation of polycystic ovary syndrome and ovarian torsion. The present work represents, to the best of our knowledge, the most up-to-date comprehensive review of the literature on the occurrence of ovarian/adnexal torsion in patients with polycystic ovaries.

The majority of ovarian torsion cases occurs in women of reproductive age, whereas postmenopausal or premenarchal women are less likely to experience it. In the present review, we present the cases of adnexal torsion in twelve patients with polycystic ovaries aged between 8 and 37 years old (mean age at diagnosis: 25 years old). Interestingly, of the twelve reported cases, only one patient was premenarchal and had experienced a total of five adnexal torsions bilaterally, while there was no reported case of ovarian or adnexal torsion in postmenopausal women with polycystic ovaries.

As far as symptomatology is concerned, most women seem to present with severe lower abdominal pain and even signs of acute abdomen. The pain is mostly acute and may even lead to vegetative symptoms such as nausea or vomiting. In cases of left-sided acute pain, diverticulitis always needs to be excluded, whereas appendicitis constitutes the most important exclusion diagnosis in the context of acute right-sided lower abdominal pain attacks.

For diagnostic purposes, laboratory testing is always required in order to exclude acute conditions such as bleeding or fulminant infections. However, in most cases of ovarian torsion and polycystic ovary syndrome, values seem to be within normal ranges. Imaging always includes a first sonographic evaluation of the ovarian size and blood supply. If inconclusive, abdominal CT scans and/or pelvic MRI scans may endorse the diagnostic evaluation process and help exclude ovarian torsion. In cases of diagnostic uncertainty, diagnostic laparoscopy or even laparotomy need to be performed with a view to directly visualize the polycystic ovaries and identify a feared adnexal or ovarian torsion.

Emergent laparoscopy with ovarian detorsion and eventual bilateral ovariopexy in case of feared recurrence is the gold standard in the context of ovarian and/or adnexal torsion in polycystic ovary syndrome. Surgical detorsion of the ovary should be performed as soon as possible; otherwise, ovarian necrosis may occur, rendering salpingo-oophorectomy inevitable. Laparotomy for ovarian/adnexal detorsion and/or salpingo-oophorectomy needs to be performed only in cases that do not allow for a laparoscopic and, hence, less-invasive, therapeutic approach.

Interestingly enough, recurrent or even habitual ovarian torsions seem to represent a major threat in patients with polycystic ovaries. Such conditions undoubtedly represent a challenge for the operating surgeon given the complexity of each case and the lack of standardized surgical approaches to this matter. Most importantly, each recurrence may be associated with severe symptomatology; complicate the procedure; and, in the worst-case scenario, even lead to permanent dysfunction and consecutive loss of the ovary and/or the adnexa.

The nonsystematic methodology regarding the study selection is one limitation of the current review. Even though the strategy of systematic literature reviews is the most accurate in terms of relevant study detection, because of the strict rules and standards, this approach demands a specific research question that fails to cover broader topics. The eventual evidence selection bias, arising from publication bias, represents an additional limitation, given that data from statistically significant studies usually succeed in reaching publication. Furthermore, a single person performed the literature analysis, which was, in turn, conducted employing solely two databases. Lastly, eventually relevant original research articles not written in the English language were not included in the data analysis.

## 5. Conclusions

Taken altogether, ovarian/adnexal torsion can occur in patients with polycystic ovary syndrome. Acute lower abdominal pain in patients with known polycystic ovaries should not be underestimated and diagnostic assessment including transvaginal ultrasound and CT or MRI scans should be performed without delay. In case of suspected torsion, emergency laparoscopy with ovarian or adnexal detorsion needs to be performed in order to save the organ. In cases of repeated ovarian/adnexal torsions, ovariopexy or ovariectomy/adnexectomy need to be discussed with the patient in order to reduce the risk of recurrence.

## Figures and Tables

**Table 1:** Cases of patients with polycystic ovary syndrome and ovarian torsion.

Study	Age (Years)	Torsion Side	Symptoms	Diagnostic Evaluation	Therapy	Remarks
Giulini et al. [25]	31	Right ovary	Tenderness in the right lower abdominal quadrant.	Leukocytosis. Transvaginal ultrasound: enlarged right ovary within coexistent mass and small amount of fluid in the pouch of Douglas.	Laparoscopic detorsion with recoloration and a decrease in size of adnexal edema.	First report of adnexal torsion after an in vitro maturation cycle.
Gonçalves et al. [26]	31	Right ovary	Sudden right pelvic pain.	Ultrasound and MRI of the right ovary: enlarged edematous right ovary with ipsilateral abnormal ovarian enhancement color sign.	Laparoscopic detorsion with recoloration and a decrease in size of adnexal edema. Plication of the utero-ovarian ligament. Oophoropexy to the round ligament. Ultimately, unilateral right adnexectomy.	Recurrent right adnexal torsion.
Hiei et al. [27]	22	Right ovary	Rebound tenderness in the right lower abdomen.	MRI: bilateral enlarged ovaries with a right twisted and thickened peduncle.	Detorsion of the twisted ovary and drilling of the bilateral ovaries via laparotomy.	Ultrasound was consistent with the MRI findings of polycystic ovary syndrome but failed to detect the stalk conditions.
Tay et al. [28]	31	Left fallopian tube	Recurrent episodes of severe pelvic pain.	Ultrasound: suspected hydrosalpinx and a small amount of free fluid.	Diagnostic laparoscopy.	Isolated fallopian tube torsion in a patient with known polycystic ovarian syndrome.
Matsuoka et al. [29]	37	Both ovaries	1. Left lower abdominal pain; 2. Right lower abdominal pain.	1. Plain CT: ovarian swelling, MRI: left ovary exhibited edematous swelling located superior–anterior to the uterus with partial cystic changes; 2. CT: mass with uneven internal absorption anterior to the uterus,	1. Laparoscopic left adnexectomy; 2. Right adnexectomy via laparotomy.	Ultrasonography could not identify either of the ovaries. Bilateral ovarian torsion.
Murakami et al. [30]	21	Right ovary	Right lower abdominal pain.	ultrasonography: enlarged right ovary, MRI: ischemic edema of the right ovary.	Emergency laparoscopy with right salpingo-oophorectomy.	Ovarian torsion associated with cessation of hormonal treatment for polycystic ovarian syndrome.
Obut et al. [31]	21	Left ovary	Pain in the lower left quadrant of the abdomen.	Ultrasound: enlarged left ovary with diminished blood perfusion.	Laparoscopy with folding and fixation of the utero-ovarian ligament to the round ligament.	Seventh recurrence of left ovarian torsion.
Pryor et al. [32]	29	Right ovary	Nausea, vomiting, and right lower quadrant pain.	Positive serum pregnancy test, leukocytosis, and enlarged right ovary in ultrasound.	Cyst drainage and detorsion by pelviscopic technique and subsequent laparotomy with right salpingo-oophorectomy.	Adnexal infarction after conservative surgical management of torsion of a hyperstimulated ovary in a pregnant patient.
Sheizaf et al. [34]	8	1. Right ovary; 2. Left ovary; 3. Left ovary; 4. Left ovary; 5. Left ovary.	1. Cramping right abdominal pain and vomiting; 2. Left abdominal pain; 3. Left abdominal pain; 4. Left abdominal pain; 5. Left abdominal pain.	1. Ultrasound: abundant free fluid and a cystic mass in the pelvis, CT scan: suggestive of right ovarian torsion; 4. Ultrasound: enlarged left ovary with no significant blood flow.	1. Laparoscopic detorsion; 2. Laparoscopic detorsion; 3. Laparoscopic detorsion with bilateral plication; 4. Laparoscopic detorsion with bilateral plication; 5. Laparoscopic detorsion with oophoropexy.	Recurrence after two oophoropexies in a prepubertal girl.
Shi et al. [35]	34	Left ovary	Left lower quadrant abdominal pain.	Leukocytosis, ultrasound imaging, and CT: enlarged ovaries with multiple follicles.	Laparoscopic detorsion.	Persistent megalocystic ovaries after ovarian hyperstimulation syndrome in a postpartum patient.
Shukunami et al. [36]	19	Right ovary	Acute abdomen and hemorrhagic shock.	Ultrasound: cystic mass in the right side of the uterus.	Emergency laparotomy with right salpingo-oophorectomy.	Twisted paraovarian cyst together with an ipsilateral polycystic ovary.
Simsek et al. [37]	20	Right ovary	Right lower quadrant tenderness and rebound tenderness.	Ultrasonography: enlarged right ovary with minimal pelvic fluid; Doppler investigation: absence of blood flow to the right ovary.	Laparoscopic detorsion and ovariopexy.	Repeated ovariopexy failure in recurrent adnexal torsion.

## Data Availability

Not applicable.
